# Potential Roles of Antiphospholipid Antibodies in Generating Platelet-C4d in Systemic Lupus Erythematosus

**DOI:** 10.3390/antib6030009

**Published:** 2017-07-02

**Authors:** Chau-Ching Liu, Travis Schofield, Amy Tang, Susan Manzi, Joseph M. Ahearn

**Affiliations:** Lupus Center of Excellence, Autoimmunity Institute, Allegheny Health Network Research Institute, Allegheny Health Network, Pittsburgh, PA 15212, USA; txs101TSS@aol.com (T.S.); amy.tang@ahn.org (A.T.); susan.manzi@ahn.org (S.M.); joseph.ahearn@ahn.org (J.M.A.)

**Keywords:** antiphospholipid antibodies, anti-cardiolipin antibodies, anti-β_2_ glycoprotein I antibodies, complement, platelet, systemic lupus erythematosus, cell-bound complement activation products (CB-CAPs)

## Abstract

Premature, accelerated onset of atherothrombotic disease is prevalent in patients with systemic lupus erythematosus (SLE). Most, if not all, atherothrombotic diseases are likely to involve platelets and complement. Previously, we discovered that platelets bearing complement activation product C4d (P-C4d) are present in SLE patients, and are significantly associated with antiphospholipid (aPL) antibody positivity and stroke in SLE patients. The goal of the present study was to further elucidate the role of aPL and other platelet-reactive autoantibodies in the generation of P-C4d. To determine the association between P-C4d and aPL antibodies, the serum levels of aPL antibodies and P-C4d of 180 SLE patients were measured by enzyme-linked immunoassays and flow cytometry, respectively. To investigate the role of aPL antibodies, and possibly other autoantibodies as well, in mediating the generation of P-C4d, in vitro 2-step P-C4d induction experiments were performed. The results showed that the presence and levels of aPL antibodies in the serum were specifically elevated in SLE patients with positive P-C4d. The plasma and immunoglobulins purified from SLE patients who were positive for P-C4d and aPL were capable of inducing C4d deposition on normal platelets in vitro. The capacity of SLE plasma in inducing P-C4d appeared to correlate proportionately to the serum aPL levels. Collectively, the results demonstrate that both aPL and other platelet-reactive autoantibodies may participate in mediating the generation of P-C4d in SLE patients.

## 1. Introduction

Premature, accelerated onset of atherosclerosis and thrombotic disease is prevalent in patients with systemic lupus erythematosus (SLE), a systemic autoimmune inflammatory disease characterized by autoantibody production, complement activation, and a myriad of clinical manifestations [1,2,3,4]. A continuously expanding spectrum of autoantibodies, including antiphospholipid antibodies, has been identified in SLE [5]. Antiphospholipid (aPL) antibodies encompass a heterogeneous group of antibodies with reactivity to various anionic phospholipid-binding proteins, including, primarily, anti-cardiolipin (aCL) antibodies, anti-β_2_ glycoprotein I (aβ_2_GPI) antibodies, and lupus anticoagulants [6,7,8,9,10]. In SLE, 30–40% of patients are found to be positive for aPL antibodies at some point of the disease course [11]. aPL antibodies have been shown to be associated with clinical events such as arterial and venous thrombosis and pregnancy complications in patients with antiphospholipid syndrome (APS) or SLE-associated APS (SLE/APS) [12,13,14,15,16].

Although the pathogenic roles of aPL antibodies in promoting thrombosis are not fully understood, abundant evidence suggests that these antibodies may function through disruption of the anticoagulant shield on cell surfaces, increase of oxidative stress, and activation of cells such as endothelial cells, monocytes, and platelets [17,18,19,20,21,22]. For example, aPL antibodies prepared from patients with APS or SLE/APS have been shown to bind to and activate platelets in vitro and in animals [23,24,25,26,27,28]. aPL antibodies may also trigger thrombosis and tissue injury via activation of the complement system [29]. In vivo studies using murine models have implicated the activation of the classical complement pathways in thrombosis and fetal loss associated with APS [30,31,32]. In humans, increased levels of complement activation products have been demonstrated in sera of patients with aPL antibodies who developed ischemic strokes [33].

We previously reported the presence of complement activation product C4d on the surface of platelets (platelet bound-C4d; P-C4d) in 18% of SLE patients, and identified a significant association between P-C4d positivity, aPL antibody positivity [34], and history of neurological manifestations in SLE patients (unpublished observations). In a recent study, P-C4d positivity was found to be significantly associated with stroke and all-cause mortality in SLE patients [35]. Other investigators have also identified associations between increased C4d deposition on platelets, and both arterial and venous thrombotic complications in patients with SLE [36,37,38]. In addition, P-C4d positivity was detected in 10% of patients with acute ischemic stroke without evidence of autoimmune disease, and correlated with stroke severity [39]. These observations, taken together, suggest an intriguing link between platelets, complement, aPL antibodies, and thrombotic disease in SLE.

Based on the studies outlined above, we hypothesize that in situ autoantibody (e.g., aPL)-mediated activation of the complement system generates C4d that can bind to platelet surfaces, and predispose platelets to a pro-thrombotic and pro-coagulating state, thereby promoting the development of thrombotic complications in patients with SLE. As a first step in verifying this hypothesis, we conducted an in-depth analysis of the prevalence and correlation of P-C4d and aPL antibodies in a cross-sectional study of 180 SLE patients, and investigated the role of aPL antibodies and other potential platelet-reactive autoantibodies in mediating C4d deposition on platelets in vitro.

## 2. Materials and Methods

### 2.1. Study Participants and Blood Specimens 

All study participants were 18 years of age or older and provided written informed consent that was approved by the institutional review board. One hundred and eighty patients who met the American College of Rheumatology (ACR) 1982 or 1997 revised classification criteria for definite SLE (≥4 criteria) [40,41] were recruited for this study during routine visits to the outpatient clinic of the Lupus Center of Excellence of the Allegheny Health Network, from July 2011 through June 2014. Two patients, who were hospitalized for disease flare and subsequently followed up at the outpatient clinic, were studied serially (up to December 2016). In addition, healthy volunteers were recruited to donate blood samples for platelet and serum preparation. 

### 2.2. Plasma, Serum, and Immunoglobulin Preparation

At the time of each participant’s visit, blood was collected into a Vacutainer tube containing ethylenediaminetetraacetic acid (EDTA) as an anticoagulant (for preparation of plasma and platelets) or a Vacutainer without anticoagulant (for serum preparation) (Becton Dickinson, Franklin Lakes, NJ, USA), and processed within 2 h after collection. An aliquot of the EDTA-anticoagulated whole blood was immediately used for P-C4d measurement. Plasma and sera were fractionated by centrifugation at 1600× *g* for 10 min and stored at 4 °C (for immediate use) or −80 °C (for later use). Patient sera were used for measuring aPL antibodies. 

Normal human serum prepared from the blood of healthy volunteers was aliquoted and stored at −80 °C for use in the in vitro P-C4d induction assays (see below). Immunoglobulin G (IgG) present in the plasma was isolated using a Pierce ImmunoPure^®^ (A/G) IgG purification kit (Thermo Scientific, Rockford, IL, USA) following the manufacturer’s instruction. After collection of IgG, the flow-through from the protein A/G affinity column was further fractionated to enrich for IgM using the Pierce Nab™ Protein L Spin purification kit (Thermo Scientific), following the manufacturer’s instruction. The IgG and IgM fractions eluted from the respective affinity column were desalted, buffer-changed into phosphate-buffered saline (PBS), and concentrated by centrifugation using Microcon^®^ centrifugal filters (molecular weight cutoff 30 kD; EMD Millipore, Billerica, MA, USA). To deplete Ig, the plasma sample was passed sequentially through the protein A/G column and protein L column twice. The final flow-through was collected, dialyzed against PBS, and concentrated back to the original volume using the Microcon^®^ device. To deplete platelet-reactive autoantibodies, the SLE plasma sample (50 µL) was incubated with platelets (approximately 10^9^) isolated from healthy volunteers at 4 °C for 30 min, and recovered by removal of platelets by centrifugation. 

### 2.3. Flow Cytometry of P-C4d Measurement 

C4d deposited on the surface of platelets (P-C4d) was measured by immunofluorescence staining/flow cytometry, as previously described [34]. Briefly, EDTA-anticoagulated whole blood was diluted in PBS prior to analysis. Platelets were electronically gated by forward scatter properties and expression of a platelet-specific marker, cluster of differentiation marker 42b (CD42b). Amounts of C4d present on platelets were assessed by using a monoclonal anti-C4d antibody (Quidel, San Diego, CA, USA) labeled with Alexa Fluor-488 using a Zenon mouse IgG1 labeling kit (Molecular Probes, Eugene, OR, USA). After staining, cells were analyzed using a FACS Calibur™ flow cytometer and Cell Quest Pro software (Becton Dickinson Immunocytometry Systems, San Jose, CA, USA). To ensure the specificity of P-C4d detected, blood aliquots from each patient stained with mouse IgG1 isotype were routinely included in all experiments. All monoclonal antibodies and Ig isotype control were used at a concentration of 5 µg/mL. Levels of P-C4d were expressed as specific median fluorescence intensity (SMFI), which was calculated as the C4d-specific median fluorescence intensity minus the isotype control median fluorescence intensity. P-C4d was considered positive based on the cut-off point of 2.15 as previously reported [34], which was derived from repeated measures of P-C4d in 100 healthy individuals.

### 2.4. aPL Antibody Immunoassays

Serum levels of aPL antibodies were determined in blood samples collected at the same time for P-C4d measurement. Serum samples of individual patients were prepared within 2 h of blood collection and stored at −80 °C until analysis. Levels of isotype-specific anti-cardiolipin (aCL) and anti-β_2_ glycoprotein I (aβ_2_GPI) antibodies in the serum were determined using the EL-aCL™ (IgM-IgG-IgA) ELISA kit and EL-β_2_GPI™ (IgM-IgG-IgA) ELISA kit (TheraTest Labs, Lombard, IL, USA), respectively, following the manufacturer’s instruction. Results are shown as standard MPL, GPL, and APL units. Positive levels for all aPL antibodies were defined following normalization to appropriate assay calibrators supplied by the manufacturer. The presence or absence of lupus anticoagulants was not assessed at the time of study.

### 2.5. In Vitro P-C4d Induction Assay

The capacity of autoantibodies in the plasma of SLE patients to active the complement system and induce C4d deposition of platelets was assessed in vitro. Briefly, platelet-rich plasma was prepared by centrifugation of EDTA-anticoagulated blood obtained from healthy volunteers or P-C4d-negative SLE patients, at 120× *g* for 10 min. Platelets were then collected and washed twice with PBS by centrifugation at 1600× *g*, in the presence of 1 g/mL of prostaglandin E1 (Sigma Aldrich, St. Louis, MO, USA). The resulting platelets were fixed with 0.5% paraformaldehyde in PBS for 20 min, washed once with PBS, and resuspended in PBS. Aliquots of platelet suspension were incubated with plasma samples (at 20% final concentration) prepared from selected SLE patients who had been identified as having positive P-C4d or high aPL serum levels. In some experiments, the SLE plasma was replaced with different amounts of purified IgG or IgM, Ig-depleted plasma, platelet-preabsorbed plasma, or commercially available human aCL antibody (Immunovision). After incubation at 4 °C for 45 min to allow autoantibody binding, the platelet samples were washed with PBS twice, resuspended in 50 µL of GVB^2+^ buffer (1% gelatin, 5 mM Na veronal, 142 mM NaCl, pH 7.3, containing Ca^2+^ and Mg^2+^), and incubated with 10 mL of normal human serum (as a source of complement). After incubation at 37 °C for 1 h to allow complement activation, the platelet suspensions were washed with PBS twice and subjected to P-C4d measurement by flow cytometry, as described above. 

### 2.6. Statistical Analysis 

Data were presented as mean and standard deviation (SD), or median and interquartile range (IQR), for continuous variables based on their distributions, and as frequency and percentage for categorical variables. Baseline comparisons of continuous variables between SLE patients with or without aPL antibodies were performed using two-sample *t* or Wilcoxon rank-sum (Mann–Whitney) tests, as appropriate. Categorical variables were analyzed using or Chi-Square or Fisher’s Exact tests. All *p-*values were considered significant at *p* < 0.05. All analyses were performed using the STATA/SE version 11.0 for Windows (Stata Corporation, College Station, TX, USA). Nonparametric technique (permutation test) was used to perform analysis of one-way multivariate data (IgA, IgM, and IgA) with approximations for ANOVA Type, Wilks’ Lambda, Lawley Hotelling, and Bartlett–Nanda–Pillai Test statistics. Multiple testing algorithms were used to control the familywise error rate [42].

## 3. Results

### 3.1. Patient Characteristics

The demographics and clinical characteristics of the 180 patients with SLE are shown in Table 1. Thirty-four patients (18.9%) were found to be P-C4d-positive (P-C4d levels >2.15) at the study visit, consistent with our previous findings [34,35]. Compared to P-C4d-negative patients (*n* = 146), P-C4d-positive patients are more likely to have a history of hematologic involvement (hemolytic anemia, lymphopenia, and thrombocytopenia) and aPL positivity (aCL and lupus anticoagulants). A significantly higher percentage of P-C4d-positive patients were on anticoagulant therapy than were P-C4d-negative patients (warfarin, *p* = 0.005; heparin, *p* = 0.025), suggesting that P-C4d-positive patients were more likely to have experienced, or were considered to be at increased risk for, thrombotic complications. An increased fraction of P-C4d-positive patients (73.5% vs. 57.5% of P-C4d-negative patients; *p* = 0.074) received steroid treatment at the time of visit, suggesting possibly higher disease activity in these patients.

### 3.2. Relationships between P-C4d and aPL Antibodies

In a previous cross-sectional study, P-C4d positivity was found to be significantly associated with aPL positivity (specifically, lupus anticoagulants, aCL IgG, and aCL IgM) [34]. The present study was aimed at further examining the prevalence of aPL antibodies and their correlation with P-C4d in SLE patients. Using commercial immunoassay kits, levels of isotype-specific aCL and aβ_2_GPI antibodies in the serum samples obtained concomitantly with P-C4d measure were determined (Table 2). In the present SLE patient cohort, 26.7% (48/180) were positive for aPL (aCL and/or aβ_2_GPI) antibodies at the study visit (Table 2). Notably, the P-C4d-positive subgroup had a considerably higher rate of aPL positivity than the P-C4d-negative subgroup (58.5% vs. 19.2%; *p* < 0.001). Compared to P-C4d-negative patients, P-C4d-positive patients were significantly more likely to have higher levels of aPL antibodies in 4 of the 6 isotypes studied (aCL IgG, aβ_2_GPI IgG, aβ_2_GPI IgM, and aβ_2_GPI IgA). Although the positivity rates of aCL IgM and aCL IgA were significantly increased in P-C4d-positive patients than in P-C4d-negative patients (both *p* = 0.012), the serum levels of these two aPL antibodies were not significantly higher in P-C4d-positive patients (*p* = 0.068 and *p* = 0.053, respectively). Of the 48 patients positive for aPL (any aCL or aβ_2_GPI isotype), 41.9% (20/48) had elevated levels of aPL antibodies of multiple isotypes (range: 2–6) at the study visit (Table 3). Notably, P-C4d-positive patients were more likely to have multiple isotypes of aPL antibodies than P-C4d-negative patients. For example, 8 out of 13 P-C4d-positive patients who had IgA aCL or aβ_2_GPI also had IgG and/or IgM aCL or aβ_2_GPI, whereas only 1 out of 8 P-C4d-negative patients who had IgA aCL or aβ_2_GPI also had IgG aCL. This finding is consistent with the fact that IgA is incapable of inducing complement activation via the classical pathway, but the concomitant presence of IgG and/or IgM aPL antibodies may be responsible for inducing complement activation on platelets. Moreover, aPL-positive-patients had significantly higher levels of P-C4d than did aPL-negative-patients (*p* = 0.002). Among the aPL-positive-patients, the P-C4d levels were differentially elevated in patients with aCL antibodies alone or aβ_2_GPI antibodies alone, and were markedly elevated in those positive for both aCL and aβ_2_GPI antibodies (Table 4).

### 3.3. Involvement of aPL Antibodies in P-C4d Generation

**Figure 1:**
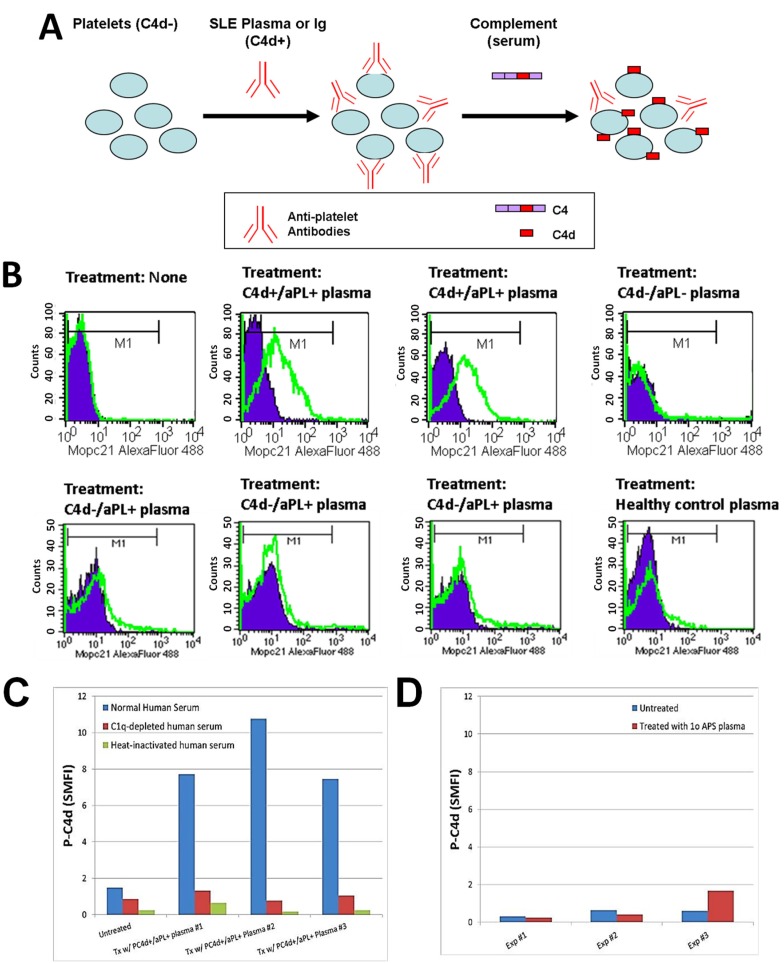
Induction of C4d deposition on the surface of platelets in vitro. (**A**) Schematic illustration of the in vitro P-C4d induction experiments. C4d-negative (C4d−) cells are incubated with plasma or Ig isolated from C4d-positive (C4d+) SLE patients, and subsequently with normal human serum (complement). After treatment, levels of C4d deposited on platelet surfaces were analyzed by flow cytometry; (**B**) Platelets from a healthy individual were incubated with the plasma of two P-C4d+/aPL+ SLE patients, one P-C4d−/aPL− SLE patient, three P-C4d−/aPL+ SLE patients, and one healthy control, followed by incubation with complement. Note the high levels of C4d deposited on cell surfaces after treatment with P-C4d+/aPL+ SLE plasma, but not with P-C4d−/aPL− SLE plasma, P-C4d−/aPL+ SLE plasma, or healthy control plasma. Purple histogram: isotype control; green open histogram: anti-C4d; (**C**) C4d deposition was induced in vitro only when platelets were treated with P-C4d+/aPL+ plasma, followed by normal human serum. C1q-depleted or heat-inactivated human serum was incapable of inducing C4d deposition; (**D**) Plasma of patients with primary antiphospholipid syndrome was incapable of inducing C4d deposition on platelets in vitro. P-C4d: platelet-bound C4d; aPL: antiphospholipid antibodies; P-C4d+/aPL+: P-C4d positive/aPL positive; P-C4d−/aPL−: P-C4d negetive/aPL negative; P-C4d−/aPL+: P-C4d negetive/aPL negative; SLE: systemic lupus erythematosus.

The observed concomitant presence of abnormal levels of aPL antibodies and P-C4d in SLE patients, together with the literature reporting binding of aPL antibodies to platelets [26,27,43], suggests that aPL antibodies constitute a major category of platelet-reactive autoantibodies and are involved in the generation and deposition of C4d on platelets in SLE patients. Therefore, we investigated whether the plasma of SLE patients contains aPL antibodies (and perhaps other autoantibodies reactive to platelets) that can bind to and induce C4d deposition on platelets in vitro. To circumvent potential confounding effects resulting from hypocomplementemia and medication (e.g., heparin) in SLE samples, and to provide an equally sufficient amount of complement in all experiments, we designed a 2-step protocol. Platelets were first incubated with SLE plasma to allow binding of antibodies, washed, and then incubated with a fixed volume of normal human serum to allow for complement activation (Figure 1A). As represented in Figure 1B, platelets derived from healthy individuals acquired significant levels of C4d on their surface after being incubated with plasma prepared from SLE patients who were tested positive for aPL antibodies and had C4d deposited on platelets ex vivo (C4d+/aPL+). In contrast, platelets were not bound by C4d after being incubated with plasma prepared either from SLE patients who had no detectable aPL antibodies or C4d on platelets ex vivo (C4d−/aPL−), or from healthy controls. Interestingly, plasma samples prepared from SLE patients who were aPL-positive but had no detectable level of P-C4d (C4d−/aPL+) were found incapable of inducing C4d deposition in vitro. When a complement component 1q (C1q)-depleted human serum or heat-inactivated normal human serum was used as the source of complement in the assay, the capacity of SLE plasma to induce P-C4d was lost (Figure 1C). In our previous study, we did not find C4d deposition on platelets of patients with primary APS ex vivo [34]. Similarly, the plasma prepared from a representative patient with primary APS was unable to induce P-C4d in vitro (Figure 1D).

**Figure 2:**
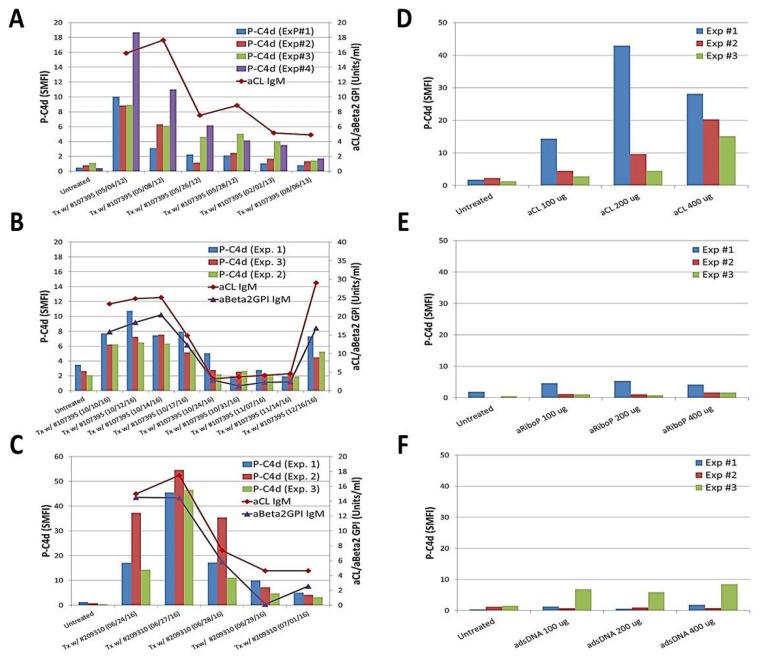
Participation of anti-phospholipid antibodies in generating the P-C4d phenotype in vitro. (**A**–**C**) Platelets prepared from healthy controls were untreated, or treated with serial plasma samples collected from an SLE patient (#107395) who was positive for aCL IgM and aβ_2_GPI IgM antibodies during two longitudinal follow-up periods during 2012–2013 (panel A) and 2016 (panel B), respectively. Plasma samples serially collected from another SLE patient (#209310) who was positive for aCL IgM and aβ_2_GPI IgM antibodies were similarly tested (panel C); (**D**–**F**) Platelets prepared from a healthy control or SLE patient were untreated or treated with a commercially available human aCL antibody (panel E), anti-ribosomal P antibody (panel E), or anti-double stranded DNA (dsDNA) antibody (panel F). Note that P-C4d was induced in a dose-dependent manner by aCL antibody, but not by anti-ribosomal P or anti-dsDNA antibody. Results shown are the mean and standard deviation derived from three independent experiments.

To further investigate the role of aPL antibodies in mediating P-C4d generation, plasma samples were serially collected from an SLE patient (#107395) whose serum aPL levels decreased over a 15-month period during 2012–2013. The capacity of these plasma samples to induce C4d deposition correlated with the aPL levels in the plasma (Figure 2A). Such correlation was noted again when she had a subsequent episode of SLE/APS flare in 2016 (Figure 2B). A similar observation was made in another SLE patient (#209310) (Figure 2C). Moreover, a commercially available human aCL IgG antibody (ImmunoVision, Inc., Springdale, AR, USA) was capable of inducing C4d deposition on platelets in a dose-dependent manner (Figure 2D). In comparison, human anti-ribosomal P (Figure 2E) and anti-dsDNA (Figure 2F) antibodies (ImmunoVision) did not induce P-C4d in vitro. Collectively, these results provide convincing support for a pivotal role of aPL antibodies in mediating generation and deposition of C4d on platelets.

### 3.4. Potential Involvement of Other Platelet-Reactive Autoantibodies in P-C4d Generation

**Figure 3:**
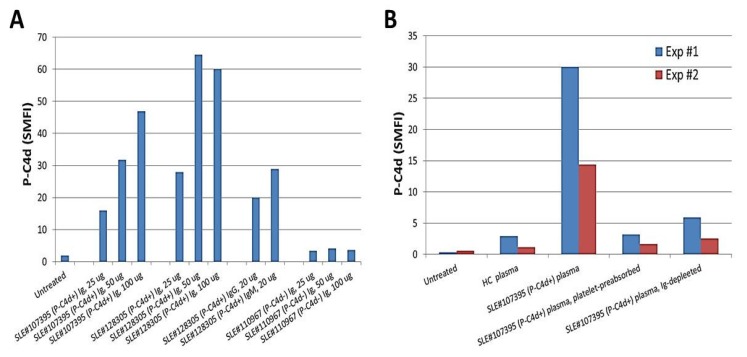
Presence of platelet-reactive autoantibodies in plasma of SLE patients. (**A**) Platelets from a healthy control were incubated with different amounts of immunoglobulins purified from two representative P-C4d+/aPL+ SLE patients (#107395 and #128305), and from a representative P-C4d-/aPL− SLE patient (#110967). P-C4d levels induced were determined by flow cytometric analysis. Note that Ig prepared from the P-C4d+/aPL+ patients induced P-C4d in a dose-dependent manner; (**B**) Platelets derived from two healthy individuals (Exp#1 and Exp#2) were treated with plasma samples derived from a healthy control (HC) or from a representative P-C4d+/aPL+ SLE patient (#107395). The SLE plasma sample was further depleted of platelet-reactive autoantibodies by pre-absorption with platelets, or depleted of immunoglobulins (Ig) using protein A/G/L.

To date, we have tested and compared plasma samples derived from different SLE patients for their capacity to induce generation of P-C4d in vitro. Overall, the majority of aPL-positive plasma samples were able to induce P-C4d efficiently, and most aPL-negative plasma samples were unable to induce P-C4d (see Figure 1B and Table 5 for representative data). However, exceptional cases existed. In some cases, plasma samples derived from SLE patients who had elevated P-C4d but otherwise tested negative for aPL antibodies were found capable of inducing C4d deposition on platelets of some SLE patients in vitro (Table 5; SLE patient #4). This finding suggested that antibodies other than aPL may react to platelets and mediate complement activation in situ on the surface of platelets. We next performed in vitro P-C4d induction experiments using Ig purified from the plasma of SLE patients. The results demonstrated that Ig purified from the plasma of SLE patients with elevated P-C4d levels and aPL antibodies were capable of inducing P-C4d deposition on platelets in vitro, while Ig purified from SLE patients with negligible P-C4d and aPL antibodies could not induce P-C4d in vitro (Figure 3A). Moreover, depletion of Ig from SLE plasma completely abolished its capacity to induce complement activation and C4d deposition on platelets in vitro (Figure 3B). This capacity of the SLE plasma was significantly decreased if the potential platelet-reactive antibodies were removed by pre-incubation of the plasma with platelets (Figure 3B). This latter result indicates that antibodies reactive to platelets are responsible for mediating the generation of P-C4d.

## 4. Discussion

We have previously demonstrated that complement activation products, particularly C4d, bind at high levels to circulating cells in patients with SLE, thereby generating a cell-bound complement activation product (CB-CAP) signature that is highly sensitive and specific for a lupus diagnosis [44,45]. Until recently, the mechanism(s) responsible for generating the CB-CAP signature have not been systematically investigated. Our recent study has shown that anti-lymphocyte autoantibodies play a pivotal role in generating patient-specific T-cell-bound C4d (T-C4d) signatures [46], providing solid evidence for an autoantibody-mediated mechanism underlying CB-CAP generation for the first time. Of the various CB-CAP phenotypes identified to date, platelet-bound C4d (P-C4d) is characteristically distinct in that it is identified only in a relatively small fraction of SLE patients (approximately 20% in sensitivity), yet in an extremely exclusive manner (99% in specificity) [34]. P-C4d was shown to be significantly associated with all-cause mortality and ischemic stroke in SLE patients [35]. Other investigators using a similar but experimentally distinct approach have observed that deposition of complement proteins C1q, C4d, and C3d are increased on platelets of patients with SLE (~48% sensitivity), and to a lesser extent, on platelets of patients with other autoimmune diseases [37,38]. Interestingly, those investigators also reported that increased C4d deposition on platelets is associated with vascular events in patients with SLE [36,37,38]. Collectively, these studies support a pathogenic role of P-C4d in a subset of SLE patients at increased risk for thrombotic events.

Given the unique features of P-C4d, the present study is focused specifically on the mechanism underlying P-C4d generation. Here we provide several lines of evidence supportive of the role of aPL antibodies and platelet-reactive autoantibodies in the generation of P-C4d. These include: (1) P-C4d-positive SLE patients had not only a higher frequency but also significantly elevated serum levels of aCL and aβ_2_GPI antibodies; (2) plasma of SLE patients was capable of inducing C4d deposition on platelets, in vitro, in an aCL/aβ_2_GPI concentration-dependent manner; (3) purified aCL antibody and Ig derived from SLE patients with aPL antibodies could induce C4d deposition on platelets in vitro; (4) plasma prepared from some SLE patients without aPL antibodies was also capable of inducing C4d deposition on platelets in vitro; and (5) this capacity was abolished by pre-absorption of the plasma with platelets.

In the inaugural study of P-C4d as a biomarker for SLE [34], it was reported that P-C4d was significantly associated with positivity for lupus anticoagulants and IgG/IgM aCL antibodies. This initial observation prompted us to speculate that aPL antibodies may be important participants in mediating the P-C4d phenotype in SLE patients. We sought to further elucidate the role of aPL antibodies through the present cross-sectional study, in which P-C4d and aPL antibody levels of a given patient were measured on the same study date, and plasma samples with known aPL antibody concentrations were used for in vitro P-C4d induction experiments. Because the presence and levels of aPL antibodies tend to wax and wane over time, this approach allows for a more definite investigation of aPL antibodies, compared to the use of historical data to indicate the aPL status of a given patient sample tested. Indeed, one patient in our SLE cohort was treated twice for a disease flare and followed longitudinally over a period of four years (2012–2016). In each flare episode, her aPL antibody levels decreased following treatment and, in parallel, the capacity of her plasma to induce P-C4d generation in vitro decreased (Figure 2A,B). Similar observations were made in another patient followed in a prospective manner (Figure 2C). The 2-step in vitro P-C4d induction assay used in the present study also allows us to decipher the complement pathway mediated by aPL antibodies. When a C1q-depleted human serum or heat-inactivated normal human serum was used as the source of complement in the assay, the capacity of SLE plasma to induce P-C4d was lost (Figure 1C). Taken together, these results provide convincing support for the involvement of aPL antibodies in mediating the generation and deposition of C4d on platelets through activation of the complement classical pathway.

In the present study, 28 SLE patients were determined to be aPL-positive yet P-C4d-negative at the study visit. Plasma derived from these aPL-positive/P-C4d-negative patients was unable to induce C4d deposition on platelets in vitro (Figure 1B). Compared to SLE patients who were positive for both aPL and P-C4d, aPL-positive/P-C4d-negative SLE patients appeared to have lower (albeit within the “positive” range) levels of aPL antibodies, and were less likely to test positive for multiple isotypes of aPL antibodies. Six of the aPL-positive/P-C4d-negative patients had only the IgA isotype of aβ_2_GPI antibody, and 2 patients had only the IgA isotype of aCL antibody, an isotype incapable of inducing complement activation via the classical pathway. Five and thirteen patients were positive for aCL IgG and aβ_2_GPI IgG, respectively. It is possible that IgG aPL antibodies in those patients are of the IgG2 or IgG4 subclasses that are inefficient in triggering complement activation.

The discoveries reported here support and extend those made recently by others. Lood and colleagues have demonstrated that increased levels of complement activation products, including C1q, C3d, and C4d, were detected on the surface of platelets of SLE patients, especially those with a history of venous thrombosis [37]. Using a one-step in vitro assay that is distinct from our current assay, these investigators also found that serum of SLE patients with a history of lupus anticoagulant supported deposition of C4d on platelets. In a more recent study, Lood et al. further reported that SLE patients with aPL antibodies had higher P-C4d levels than those without aPL antibodies, and that aPL antibodies can activate platelets and induce C4d deposition in vitro [38]. Peerschke and colleagues demonstrated that sera from SLE patients could fix complement and induce C1q/C4d deposition on platelets in vitro, measured by a solid phase-based enzyme-linked immunosorbent assay [36]; this complement fixation/activation activity was associated with the presence of IgG aCL and aβ_2_GPI antibodies in serum samples tested.

In the current study cohort, 41% of P-C4d positive patients had undetectable or low (below the positive cutoff) levels of aPL antibodies at the study visit (Table 2). There are two possible explanations for this apparent lack of correlation between P-C4d and aPL antibodies. First, an extremely low concentration of aPL antibodies, particularly those of the IgM isotype, may not be detectable by conventional immunoassays but may be adequate to bind to platelets and activate/generate C4d in situ on platelet surfaces. Second, it is possible that other platelet-reactive autoantibodies may participate in generating P-C4d in vivo. The latter possibility is supported by the observation that plasma of aPL-negative SLE patients was capable of inducing P-C4d in vitro (Table 5), and that removal of platelet-reactive antibodies by preabsorption of SLE plasma with platelets abolished the in vitro P-C4d-inducing activity (Figure 3B). Several platelet-reactive autoantibodies recognizing normally expressed platelet surface molecules such as glycoprotein IIb/IIIa (GPIIb/IIIa), have previously been reported [47,48]. It is also possible that SLE patients may develop autoantibodies against disease-related “neo” antigens specifically expressed on their platelets. We postulate that such autoantigens/autoantibodies may be present either generally in all SLE patients, or differentially among individual SLE patients. Indeed, the in vitro P-C4d induction experiments have shown that plasma of a group of SLE patients was capable of inducing C4d deposition on platelets derived from both healthy individuals and SLE patients (Table 5; SLE patients #1 and #3), whereas plasma from another group of SLE patients was only able to induce C4d deposition on platelets from a few selected SLE patients (Table 5; SLE patients #2 and #4). These results suggest that the former group of SLE patients may have developed autoantibodies that react with antigens normally expressed on platelets, whereas the latter group of SLE patients may have developed autoantibodies that recognize “neo” antigens expressed only on SLE platelets. Further studies are warranted to identify such potential platelet-reactive autoantibodies in SLE patients.

In summary, the present study not only advances previous findings regarding the potential role of aPL antibodies in generating P-C4d in SLE patients, but, together with our recent study on T-cell-bound C4d [46], also solidifies further the mechanistic model of autoantibody-mediated generation of CB-CAP signatures in SLE patients.

## Figures and Tables

**Table 1:** Patient demographics and clinical characteristics.

		Platelet-C4d		
	All (*n* = 180)	Positive (*n* = 34)	Negative (*n* = 146)	*p*-Value
Age, year, mean (SD)	47.2 (11.9)	46.9 (10.7)	47.3 (12.2)	0.802
Duration of SLE, year, mean (SD)	15.2 (9.7)	16.5 (9.6)	14.8 (9.7)	0.276
Sex, *n* (%)				
Women	167 (92.8)	31 (91.2)	136 (93.2)	0.714
Race, *n* (%)				
White	156 (86.7)	28 (82.4)	128 (87.7)	0.240
Black	21 (11.7)	6 (18.6)	15 (10.3)	
Others	3 (1.7)		3 (2.0)	
ACR ^&^ criteria (ever), *n* (%)				
Malar rash	83 (46.1)	13 (38.2)	70 (47.9)	0.344
Discoid rash	14 (7.8)	4 (11.8)	10 (6.8)	0.306
Photosensitivity	111 (61.7)	17 (50.0)	94 (64.4)	0.170
Oral ulcers	122 (67.8)	19 (55.9)	103 (70.3)	0.107
Arthritis	172 (95.6)	32 (94.1)	140 (95.9)	0.647
Serositis	64 (35.6)	14 (41.2)	50 (34.2)	0.551
Renal disease	54 (30.0)	8 (23.5)	46 (31.5)	0.412
Neurological	20 (11.1)	4 (11.8)	16 (10.9)	1.000
Seizure	15 (8.3)	1 (2.9)	14 (9.6)	0.310
Psychosis	9 (5.0)	3 (8.8)	6 (4.1)	0.402
Hematological	96 (53.3)	25 (73.5)	71 (48.6)	0.012
Hemolytic anemia	10 (5.6)	6 (17.6)	4 (2.7)	0.004
Leukopenia	46 (25.6)	11 (32.4)	35 (24.0)	0.382
Lymphopenia	61 (33.9)	19 (55.9)	42 (22.8)	0.004
Thrombocytopenia	32 (17.8)	10 (29.4)	22 (15.1)	0.047
Antinuclear antibody	177 (98.3)	33 (97.1)	144 (98.6)	0.468
Serological	142 (78.9)	33 (97.1)	109 (74.7)	0.002
Anti-Phospholipid ^#,^^	83 (46.1)	24 (70.6)	59 (40.4)	0.002
Anti-cardiolipin	60 (33.3)	17 (50.0)	43 (29.5)	0.028
Lupus anticoagulant	45 (25.0)	16 (47.1)	29 (19.9)	0.002
Anti-dsDNA	97 (53.9)	23 (67.6)	74 (50.7)	0.087
Anti-Smith	24 (13.3)	4 (11.8)	20 (13.7)	1.000
Medication use, current **, *n* (%)				
Steroid	109 (60.6)	25 (73.5)	84 (57.5)	0.074
Antimalarial	128 (71.1)	23 (67.6)	105 (71.9)	0.676
Anticoagulant				
Warfarin	24 (13.3)	10 (29.4)	14 (9.6)	0.005
Heparin	7 (3.9)	4 (11.8)	3 (2.1)	0.025
FXa inhibitor	2 (1.1)	1 (2.9)	1 (0.7)	0.343
Antiplatelet				
Aspirin	69 (38.3)	11 (32.4)	58 (39.7)	0.557
P2Y12 antagonist	10 (5.6)	4 (11.8)	6 (4.1)	0.096
Statins	37 (20.6)	7 (20.6)	30 (20.5)	1.000
Immunosuppressant	101 (56.1)	21 (61.8)	80 (50.8)	0.566
Biologicals	18 (10.0)	4 (11.8)	14 (9.6)	0.751
Antihypertensive	77 (42.8)	13 (38.2)	64 (43.8)	0.571
Diuretics	31 (17.2)	3 (8.8)	28 (19.2)	0.208
Insulin	6 (3.3)	0 (0.0)	6 (4.1)	0.592
Antidiabetic	4 (2.2)	0 (0.0)	4 (2.7)	1.000
P-C4d level, current, median (IQR)	0.6 (0.2–1.4)	5.6 (3.4–11.1)	0.4 (0.1–0.8)	<0.001

* Continuous variables are presented as mean (SD) or median depending on the data distribution. ^&^ ACR: American College of Rheumatology. ^#^ Eighty-three patients had a history of aPL antibody positivity prior to the current study visit. ^ Of the 83 historically aPL-positive patients, 32 remained positive at the current study visit. In addition, 16 patients without a history of aPL antibody positivity were tested positive at the current study visit (see Table 2 for further information). ** Medication use at the study visit: antimalarial—hydroxychloroquine, chloroquine, and quinacrine; immunosuppressants—azathioprine, methotrexate, mycophenolate mofetil, mycophenolic acid, cyclophosphamide, tacrolimus, and leflunomide; biologicals—belimumab. P-C4d: platelet-bound C4d; aPL: antiphospholipid. SLE: systemic lupus erythematosus. IQR: interquartile range.

**Table 2:** Comparison of serum anti-Cardiolipin (aCL) and anti-β_2_GPI (aβ_2_GPI) levels in P-C4d-positive vs. P-C4d-negative patients.

		Platelet-C4d		
	All (*n* = 180)	Positive (*n* = 34)	Negative (*n* = 146)	*p*-Value (P-C4d+ vs. P-C4d−)
aPL (aCL and/or aβ_2_GPI) antibodies positivity, *n* (%)	48 (26.7)	20 (58.5)	28 (19.2)	<0.001
aCL IgM positivity, *n* (%) ^†^	19 (10.6)	8 (23.5)	11 (7.5)	0.012
aCL IgM level (U/mL) median (IQR) *	3.1 (2.0–6.5)	4.2 (2.2–11.6)	2.8 (2.0–6.0)	0.068
aCL IgG positivity, *n* (%) ^†^	17 (9.4)	11 (32.4)	6 (4.1)	<0.001
aCL IgG level (U/mL) median (IQR) *	5.0 (2.8–9.6)	12.5 (5.6–37.9)	4.6 (2.7–7.7)	<0.001
aCL IgA positivity, *n* (%) ^†^	6 (3.3)	4 (11.8)	2 (1.4)	0.012
aCL IgA level (U/mL) median (IQR) *	1.2 (0.6–2.3)	1.3 (1.0–2.9)	1.0 (0.5–2.0)	0.053
Any aCL positivity, *n* (%)	32 (17.8)	13 (38.2)	19 (13.0)	0.002
aβ_2_GPI IgM positivity, *n* (%)	10 (5.6)	5 (14.7)	5 (3.4)	0.022
aβ_2_GPI IgM level (U/mL) median (IQR)	0.4 (0.0–1.3)	1.0 (0.4–3.6)	0.3 (0.0–0.9)	<0.001
aβ_2_GPI IgG positivity, *n* (%)	14 (7.8)	11 (32.4)	8 (5.5)	<0.001
aβ_2_GPI IgG level (U/mL) median (IQR)	1.6 (0.0–3.7)	4.6 (1.1–76.1)	1.3 (0.0–3.1)	<0.001
aβ_2_GPI IgA positivity, *n* (%)	21 (11.7)	13 (38.2)	16 (11.0)	<0.001
aβ_2_GPI IgA level (U/mL) median (IQR)	0.4 (0.0–2.6)	3.9 (0.8–12.8)	0.4 (0.0–1.2)	<0.001
Any aβ_2_GPI positivity, *n* (%)	34 (18.9)	18 (52.9)	16 (11.0)	<0.001

^†^ Positivity for all aPL antibodies was defined based on the cutoff values provided by the ELISA kits used. * median (IQR). C4d−: C4d-negative; C4d+: C4d-positive; aβ_2_GPI: anti-β_2_ glycoprotein I.

**Table 3:** Distribution of anti-Cardiolipin (aCL) and anti-β_2_GPI (aβ_2_GPI) antibodies in SLE patients.

Number of Positive aCL and aβ_2_GPI Antibodies	Number of Patients	Isotypes of Positive aCL and aβ_2_GPI Antibodies
6	1	aCL IgM/IgG/IgA; aβ_2_GPI IgM/IgG/IgA
5	1	aCL IgM/IgG/IgA; aβ_2_GPI IgG/IgA
	1	aCL IgM/IgG/IgA; aβ_2_GPI IgM/IgG
4	1	aCL IgG/IgA; aβ_2_GPI IgG/IgA
	1	aCL IgM/IgG; aβ_2_GPI IgG/IgA
3	2	aCL IgG; aβ_2_GPI IgG/IgA
	1	aCL IgM/IgG; aβ_2_GPI IgG
	1	aβ_2_GPI IgM/IgG/IgA
	1	aCL IgM/IgG/IgA
2	3	aCL IgM; aβ_2_GPI IgM
	5	aCL IgG; aβ_2_GPI IgG
	2	aCL IgM; aβ_2_GPI IgA
1	8	aCL IgM
	3	aCL IgG
	2	aCLIgA
	2	aβ_2_GPI IgM
	1	aβ_2_GPI IgG
	12	aβ_2_GPI IgA

**Table 4:** Comparison of P-C4d levels in SLE patients with or without aPL antibodies.

	P-C4d Median (IQR) *	*p*-Value (vs. aPL−)
aPL− (*n* = 132)	0.52 (0.13–0.91)	
aPL+ (*n* = 48) **	1.18 (0.25–4.55)	0.002
aCL+ alone (*n* = 14)	0.48 (0.20–1.51)	0.586
aβ_2_GPI+ alone (*n* = 16)	1.44 (0.25–3.24)	0.042
aCL+/aβ_2_GPI+ (*n* = 18)	4.61 (0.53–11.44)	0.001

* P-C4d (specific median florescence intensity; SMFI): median (interquartile range). ** aPL+: antiphospholipid antibody positive including aCL and/or aβ_2_GPI antibodies. aPL+: aPL-positive; aPL−: aPL-negative.

**Table 5:** Comparison of the capacity of aPL+ and aPL− SLE plasma to induce P-C4d in vitro.

Plasma Source ^a^	Plasma aPL Positivity ^b^	Ex vivo P-C4d Positivity ^c^	Platelet Source ^d^	In Vitro P-C4d (SMFI) ^e^
SLE patient #1	+	+	SLE patient #4	34.21
SLE patient #1			SLE patient #5	39.17
SLE patient #1			SLE patient #6	19.31
SLE patient #1			SLE patient #7	14.70
SLE patient #1			SLE patient #8	6.83
SLE patient #1			SLE patient #9	27.34
SLE patient #1			SLE patient #10	39.29
SLE patient #1			SLE patient #11	31.52
SLE patient #1			Healthy Control #1	27.50
SLE patient #1			Healthy Control #2	5.38
SLE patient #2	+	+	SLE patient #4	4.93
SLE patient #2			SLE patient #7	5.20
SLE patient #2			SLE patient #8	0.44
SLE patient #2			SLE patient #9	6.20
SLE patient #2			SLE patient #10	9.37
SLE patient #2			SLE patient #11	9.53
SLE patient #2			SLE patient #12	3.36
SLE patient #2			Healthy Control #1	1.15
SLE patient #2			Healthy Control #2	0.78
SLE patient #3	+	+	SLE patient #12	20.48
SLE patient #3			Healthy Control #1	24.92
SLE patient #3			Healthy Control #2	14.30
SLE patient #4	−	+	SLE patient #5	3.07
SLE patient #4			SLE patient #6	0.80
SLE patient #4			SLE patient #7	2.08
SLE patient #4			SLE patient #8	0.26
SLE patient #4			SLE patient #9	3.96
SLE patient #4			SLE patient #10	7.20
SLE patient #4			SLE patient #11	8.70
SLE patient #4			Healthy Control #1	0.10
SLE patient #4			Healthy Control #2	1.14

^a^ Plasma samples used in the first step of the in vitro P-C4d induction experiments. ^b^ Positivity for aCL and/or aβ_2_GPI antibodies in the plasma source determined by ELISA. ^d^ Positivity of P-C4d on platelets of the donor of the plasma source. ^d^ Platelets derived from healthy controls or SLE patients who have no/low C4d on platelet surfaces. ^e^ C4d levels on the surface of treated platelets, determined by flow cytometry. Boldfaced numbers indicate positive P-C4d (SMFI. 2.15). +: positivity; –: negativity.
